# Study of Thermal Properties, Molecular Dynamics, and Physical Stability of Etoricoxib Mixtures with Octaacetylmaltose near the Glass Transition

**DOI:** 10.3390/ijms23179794

**Published:** 2022-08-29

**Authors:** Katarzyna Grzybowska, Marzena Rams-Baron, Kinga Łucak, Andrzej Grzybowski, Marian Paluch

**Affiliations:** 1 Institute of Physics, University of Silesia in Katowice, ul. 75 Pułku Piechoty 1, 41-500 Chorzow, Poland; 2 Silesian Center for Education and Interdisciplinary Research, ul. 75 Pułku Piechoty 1a, 41-500 Chorzow, Poland

**Keywords:** molecular dynamics near the glass transition, physical stability of amorphous drugs against their recrystallization, amorphous binary mixtures

## Abstract

In this paper, we thoroughly investigated the physical stability of the anti-inflammatory drug etoricoxib, which has been reported earlier to be resistant to recrystallization in its glassy and supercooled states at ambient pressure. Our unique application of the standard refractometry technique showed that the supercooled liquid of the drug was able to recrystallize during isothermal experiments in atmospheric conditions. This enabled us to determine the crystallization onset timescale and nucleation energy barrier of etoricoxib for the first time. As the physical instability of etoricoxib requires working out an efficient method for improving the drug’s resistance to recrystallization to maintain its amorphous form utility in potential pharmaceutical applications, we focused on finding a solution to this problem, and successfully achieved this purpose by preparing binary mixtures of etoricoxib with octaacetylmaltose. Our detailed thermal, refractometry, and molecular dynamics studies of the binary compositions near the glass transition revealed a peculiar behavior of the glass transition temperatures when changing the acetylated disaccharide concentration in the mixtures. Consequently, the anti-plasticization effect on the enhancement of physical stability could be excluded, and a key role for specific interactions in the improved resistance to recrystallization was expected. Invoking our previous results obtained for etoricoxib, the chemically similar drug celecoxib, and octaacetylmaltose, we formulated a hypothesis about the molecular mechanisms that may cause an impediment to crystal nuclei formation in the amorphous mixtures of etoricoxib with octaacetylmaltose. The most plausible scenario may rely on the formation of hydrogen-bonded heterodimers of the drug and excipient molecules, and the related drop in the population of the etoricoxib homodimers, which disables the nucleation. Nevertheless, this hypothesis requires further investigation. Additionally, we tested some widely discussed correlations between molecular mobility and crystallization properties, which turned out to be only partially satisfied for the examined mixtures. Our findings constitute not only a warning against manufacturing the amorphous form of pure etoricoxib, but also evidence for a promising outcome for the pharmaceutical application of the amorphous compositions with octaacetylmaltose.

## 1. Introduction

In the last few decades, the role of amorphous pharmaceuticals has increased significantly in the modern pharmaceutical science and industry [1]. Their advantages over their crystalline counterparts include a much better solubility in water, and consequently a higher bioavailability that enhances the therapeutic efficiency of the amorphous drugs. However, the maintenance of the physical stability of amorphous solid pharmaceutical formulations, at least during the shelf-time required by pharmaceutical standards (i.e., a minimum of 2 years), still presents the main challenge that should be met before putting such novel medicines on the market. The encountered physical instability of amorphous drugs is naturally explained by their thermodynamically unstable glassy state, which is most often achieved as the final form of amorphous formulation. Although there are different methods for amorphization, fundamental studies of the glass transition have typically focused on the vitrification process, i.e., the quench cooling of a liquid, which subjects the liquid to supercooling and then transforms it into a glass, avoiding any crystallization. The supercooled liquid state is widely considered as very interesting from a cognitive point of view, and it is commonly believed to provide a premise for predicting the physical stability of its vitrified form. From the viewpoint of pharmaceutical applications, one flaw in the materials for both the supercooled liquid and glassy states is their usually high tendency to recrystallize due to the thermodynamic metastability of supercooled liquids and the aforementioned thermodynamic instability of glasses. Therefore, the progress already made and further expected towards the improvement of the physical stability of non-crystalline medicines has resulted from many efforts invested into studying their thermal and molecular dynamic properties and the process of recrystallization from their supercooled liquid and glassy states, in order to better understand the molecular mechanisms that can be employed in preparing sufficiently long-stable amorphous solid pharmaceuticals [1,2,3,4,5,6,7,8,9]. 

Among many anti-inflammatory agents that have undergone vitrification, etoricoxib (ETB) has stood out as having a very high resistance to recrystallization from both the supercooled liquid and glassy states at ambient pressure in all earlier investigations, by means of differential scanning calorimetry (DSC) and broadband dielectric spectroscopy (BDS) [10]. Interestingly, another chemically related anti-inflammatory agent, celecoxib (CEL), easily recrystallizes above and below the glass transition temperature *T_g_* at ambient pressure [11,12]. Similarly, rofecoxib (RFB), belonging to the same class of selective cyclooxygenase-2 (COX-2) inhibitors, has a high tendency to recrystallize [10], as earlier argued by showing its highest overall thermodynamic force to crystallization in a series of examined coxibs [13]. On the molecular level, the differences in the recrystallization tendency between ETB and other coxibs have been suggested to be the result of a tautomerization effect on the interactions between ETB molecules, favoring the formation of heterodimers between two different ETB isomers, as concluded from the density functional theory (DFT) calculations, Fourier-transform infrared (FTIR) spectroscopy, and BDS measurements [10,14]. In the opposite way, the tautomerization is not expected to occur in the case of CEL, for example, the molecules of which easily form homodimers that facilitate the crystallization process. 

However, the prevention of ETB crystallization is broken at elevated pressures. Previous reports [10,15] have shown that the isothermal compression of ETB at even small pressures (about 10–20 MPa) leads to its crystallization, which gives us a warning of possible troubles with manufacturing the amorphous ETB, which can devitrify under compression during the tableting process. The intriguing tendency to recrystallize revealed by ETB encouraged us to further investigate this anti-inflammatory drug at ambient pressure.

In this paper, we implemented a standard refractometry technique to shed new light on the crystallization tendency of pure ETB at ambient pressure. Since the physical stability of this drug has turned out to be unmaintained, we thoroughly examined a series of amorphous binary mixtures of ETB with different contents of octaacetylmaltose (acMAL), which was earlier successfully applied to stabilize the amorphous CEL [16]. Using the DSC, BDS, and refractometry measurements, which are described in detail in Section 3, we showed that the presence of acMAL in amorphous mixtures with ETB significantly increased the resistance of the amorphous drug to recrystallization. 

## 2. Results and Discussion

### 2.1. Amorphous Pure ETB: Recrystallization Study

**Figure 1:**
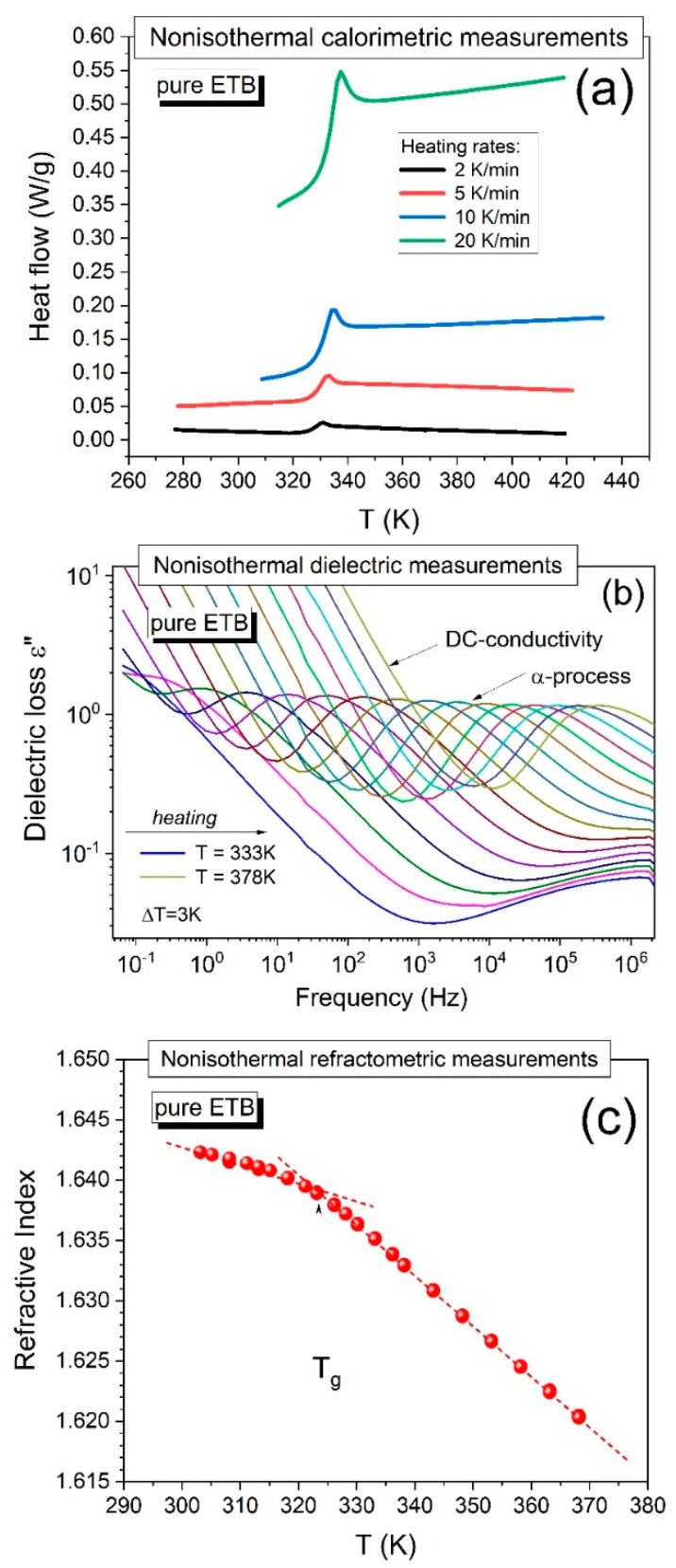
Illustration of a non-isothermal study on the physical stability conducted for pure ETB near the glass transition temperature *T_g_* by using (**a**) DSC—plots of the temperature dependences of heat flow at different experimental rates (2, 5, 10, and 20 K/min), (**b**) BDS—plots of dielectric loss spectra measured in the temperature range between 333 K and 378 K every ∆T = 3 K, and (**c**) refractometry measurements—the temperature dependence of the refractive index. The dotted lines illustrate the method for determining the value of *T_g_* at their intersection.

In the first step, we verified and extended an earlier study on the physical stability of amorphous pure ETB [10] by performing non-isothermal calorimetric measurements using various heating rates (2, 5, 10, and 20 K/min) at ambient pressure. As can be seen in Figure 1a, the earlier vitrified pure ETB did not reveal any signs of recrystallization upon heating the sample independently from the applied heating rate, even in cases of such a low experimental rate as 2 K/min, and the glass transition temperature determined at the midpoint of the jump in the heat flow typically increased (*T_g_* = 327, 328.5, 330, and 332.5 K, respectively) with increasing heating rate.

A similar picture of the physical stability of amorphous pure ETB can be concluded from the dielectric loss spectra obtained during the heating of the sample from a glassy state to a supercooled liquid state. An example of such dielectric measurement results is shown in Figure 1b, where the sample was heated from T = 333 K to T = 378 K, and the dielectric spectra were collected every 3 K. The dielectric loss spectra typically shifted to high frequencies with increasing temperature, and no sharp drops were observed in the amplitude of the structural α-relaxation time. The latter characteristic indicated that the dielectric measurements gave no evidence of sample recrystallization [2,10,15,16,17,18]. 

Herein, we additionally applied the refractometry technique to detect the tendency of pure ETB to recrystallize under non-isothermal conditions. The measurements of the refractive index on cooling ETB from its supercooled liquid to its glassy state did not show any signs of the recrystallization of the drug, which can be seen in Figure 1c, where the glass transition is well-distinguished (manifested by a change in the slope of the nearly linear temperature dependences of the refractive index measured in the supercooled liquid and glassy states), as we describe in detail in Section 2.2. 

We also made many attempts to study the isothermal recrystallization of pure ETB at ambient pressure by using DSC and BDS measurements, which resulted in no signs of devitrification. However, it was possible to disrupt the equilibrium state between the two kinds of ETB tautomers in the BDS experiments by compressing the sample under isothermal conditions, which most probably triggered its recrystallization [10,15]. A question arises as to whether one could find such experimental conditions, which would result in a similar breaking of the balance between the populations of the two ETB tautomers, consequently causing sample recrystallization at ambient pressure. At this point, it should be noted that this is not only a cognitive challenge, but also an important application problem, which could influence the manufacturing process, implementing the transition of the procedures worked out during the laboratory fundamental investigations to the larger industrial scale. For this reason, in the study of the isothermal recrystallization tendency of amorphous pure ETB at ambient pressure, we have tested the standard refractometry technique, which is characterized by a different geometry and larger size of the measurement sample compared to those used in the standard DSC and BDS experiments. In Section 3.2, Section 3.3 and Section 3.4, detailed descriptions of the different shapes and sizes of the measurement vessels and samples are provided.

**Figure 2:**
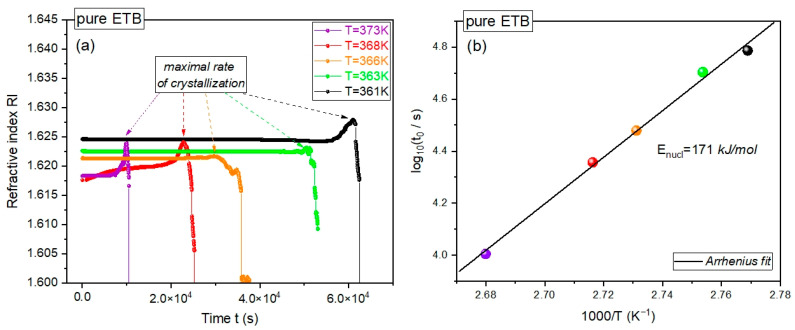
Isothermal recrystallization study of pure ETB by means of the refractometry technique. (**a**) Plots of the refractive index vs. time at the constant temperatures 361, 363, 366, 368, and 373 K. (**b**) Plot of the recrystallization onset time vs. inverse temperature. The solid line represents the linear fit of the experimental data to Equation (1), the fitted slope of which yields an activation energy for nucleation of *E_nucl_* ≈ 171 kJ/mol for pure ETB at ambient pressure.

We performed time-dependent crystallization refractometry measurements of pure ETB at several constant temperatures above *T_g_* at ambient pressure, collecting the refractive index (RI) in tens of thousands of time steps, as shown in Figure 2a. At each temperature, we observed a sharp drop in the dependence of RI on time *t*, which gives evidence for the recrystallization of the supercooled pure ETB under isothermal conditions. Such a drop has always been preceded by a well-distinguished maximum in the dependence of RI(*t*). The time *t* = *t_onset_* at which the maximum in RI(*t*) occurred can be considered as the onset time of the isothermal crystallization process, and can be reasonably interpreted as a well-established estimation of the lag time for nucleation. 

The possibility of determining the crystallization onset time or the corresponding crystallization rate enabled us to evaluate the activation energy for nucleation during the isothermal crystallization of supercooled pure ETB at ambient pressure, which has never been determined before. As depicted in Figure 2b, the dependence of the logarithm of *t_onset_* on the inverse temperature 1/*T* can be very well-fitted to a linear function that obeys the simple Arrhenius activation law:

(1) $$\log_{10}t_{onset}=\log_{10}t_{onset}^{0}+\frac{E_{nucl}}{RT}\log_{10}e$$

where *R* is the gas constant and the fitting parameters are $t_{onset}^{0}$ and $E_{nucl}$. The latter denotes the sought-after activation energy for nucleation during isothermal crystallization. By fitting the refractometry data for isothermal crystallization into Equation (1), we obtained $\log_{10}\left(t_{onset}^{0}/s\right)=-19.9\pm 0.9$ and $E_{nucl}=\left(171\pm 7\right)\mathrm{kJ}/\mathrm{mol}$, where the latter was even less than the activation energy for nucleation during the isothermal crystallization of CEL, $E_{nucl}=\left(217\pm 21\right)\mathrm{kJ}/\mathrm{mol}$, which was evaluated herein by using the BDS measurements reported in [12]. This means that the amorphous pure drugs CEL and ETB were characterized by similar energy barriers for isothermal crystallization at ambient pressure.

It is worth noting that standard refractometry has not been a typical method for studying the crystallization process until recently. To the best of our knowledge, our attempt at employing it in these isothermal crystallization investigations is probably the first systematic and successful approach to the issue, which yielded results of a similar quality to those obtained by means of DSC or BDS measurements.

Since the physical stability of amorphous pure ETB has been undermined, even at ambient pressure using refractometry measurements, it is necessary to work out an efficient method for preventing the drug’s recrystallization in order to continue considering pharmaceutical applications of its amorphous form. Taking into account the earlier successful stabilization of amorphous CEL by mixing it with acMAL [16] and the very similar molecular structures of CEL and ETB, we investigated a few amorphous binary mixtures of ETB with acMAL containing different weight fractions of the excipient, i.e., 10, 20, 30, 40, or 50 wt% acMAL. 

### 2.2. Amorphous Binary ETB—acMAL Mixtures: Thermal and Optical Properties 

**Figure 3:**
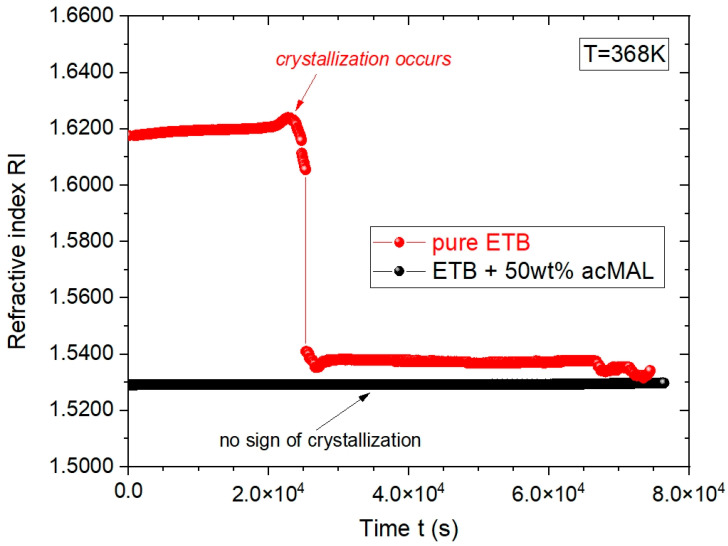
Plots of the dependences of refractive index vs. time for pure ETB (marked in red) and its mixture with 50 wt% acMAL (marked in black) at the constant temperature T = 368 K.

First, we performed comparative refractometric investigations of the tendency for recrystallization in amorphous pure ETB and its amorphous compositions with acMAL in isothermal conditions above *T_g_*, finding a considerably higher ability of the amorphous ETB–acMAL mixtures to avoid recrystallization. As an example, Figure 3 shows the time evolutions of the RI collected for pure ETB and its composition with 50 wt% acMAL at the constant temperature T = 368 K. 

One can see that the RI value for the supercooled ETB + 50 wt% acMAL mixture equaled about 1.53 at T = 368 K and remained approximately constant for the duration of the experiment, which was carried out for ca. 22 h, while pure ETB has crystallized after about 6 h. This means that the addition of acMAL to ETB significantly enhanced the resistance of the amorphous ETB composition to recrystallization.

**Figure 4:**
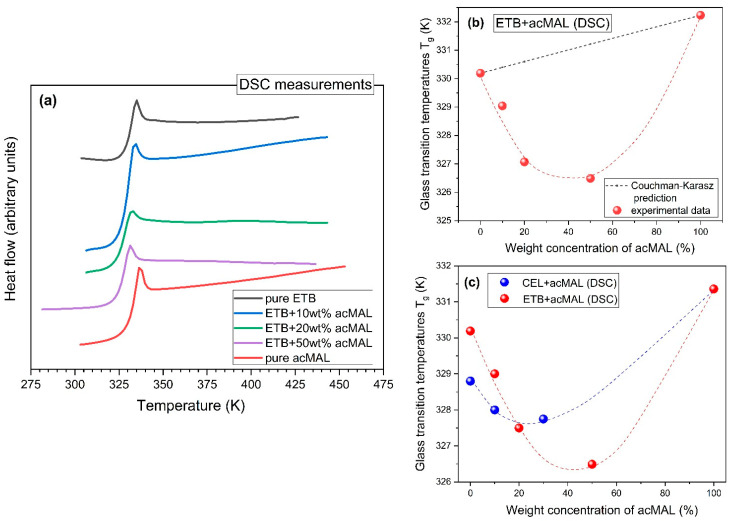
(**a**) Plot of the temperature dependences of the heat flow of the amorphous mixtures of ETB with 10, 20, and 50 wt% acMAL, obtained at the heat rate of 10 K/min, compared with those earlier reported for pure ETB [10] and pure acMAL [16]. (**b**) Plot of the calorimetric glass transition temperatures of the amorphous ETB–acMAL mixtures with different percentage weight concentrations of acMAL. The red dotted curve is a guide for the eyes and the black dotted curve was predicted from Equation (2). (**c**) Comparison of the peculiar behaviors of the dependences of *T_g_* on the content of wt% acMAL in the binary mixture established herein, from DSC measurements for the amorphous ETB–acMAL compositions and from those previously reported [16] for the CEL–acMAL mixtures. The dotted curves are guides for the eyes.

Systematic standard DSC measurements, performed during heating at a rate of 10 K/min in the case of the amorphous mixtures containing 10, 20, and 50 wt% acMAL, as well as analogous DSC experiments carried out for their amorphous pure components, revealed a jump in the heat flow, which is characteristic of the glass transition, and no sign of exothermic thermal effects that could have been classified as a recrystallization process (see Figure 4a). Thus, the non-isothermal physical stability observed earlier for amorphous pure ETB in the DSC measurements was also established for its amorphous compositions with acMAL, which is further undergoing a thorough examination by us as a good candidate for improving the physical stability of amorphous ETB, under both isothermal and non-isothermal conditions. 

By analyzing the glass transition temperatures obtained from the DSC experiments, which are shown in Table 1, a few interesting observations were made. First of all, it should be noted that the values of *T_g_* for ETB and acMAL were very close, i.e., *T_g_* = 330 K and *T_g_* = 332 K, respectively. A slightly larger value of *T_g_* for acMAL than for ETB suggested that the anti-plasticization effect should not play an important role in the molecular mechanisms of preventing the recrystallization process in the amorphous ETB–acMAL binary mixtures. In fact, the values of *T_g_* determined for these amorphous compositions were less than those established for the pure components and increased with increasing acMAL content in the mixture. This means that the acMAL even plasticized the ETB, despite exerting an undoubtedly favorable effect on the enhancement of the physical stability of amorphous ETB by playing the role of a recrystallization inhibitor. 

Such a conclusion was also supported by applying the Couchman–Karasz equation [19] to predict the glass transition temperature, *T_g_*, of the binary mixtures, which was developed from the earlier-proposed Gordon–Taylor equation [20] as follows:

(2) $$T_{g}=\frac{w_{ETB}T_{g\;ETB}+Kw_{acMAL}T_{g\;acMAL}}{w_{ETB}+Kw_{acMAL}}$$

where $w_{ETB}$ and $w_{acMAL}$ are the weight fractions of ETB and acMAL, respectively; $T_{g\;ETB}$ and $T_{g\;acMAL}$ denote the glass transition temperatures of the pure components; and $K=\Delta C_{p\;\;acMAL}/\Delta C_{p\;\;ETB}$ is a measure of the interactions between the mixture components, which can be determined from the DSC measurements by finding the jumps in the heat capacity, $\Delta C_{p\;\;ETB}$ and $\Delta C_{p\;\;acMAL}$, at the glass transition of the pure components. Assuming the values $\Delta C_{p\;\;ETB}=0.347\;J/(\mathrm{gK})$ and $\Delta C_{p\;\;acMAL}=0.350\;J/(\mathrm{gK})$, which were obtained from the calorimetric measurement data, we obtained a value of *K* equal to 1.009, which led to a nearly linear dependence based on Equation (2), while the experimental data of *T_g_* clearly diverged from the Couchman–Karasz prediction, as can be seen in Figure 4b. Since the Couchman–Karasz equation was not satisfied by the values of *T_g_* found for the amorphous ETB–acMAL mixtures, one cannot expect that the physical stability of the compositions was enhanced compared to the amorphous pure ETB, due to the anti-plasticization effect. 

A similar non-monotonic behavior, manifesting in a minimum of the dependence of the *T_g_* values on the excipient content, has been established earlier for amorphous CEL–acMAL mixtures [16]. By inspecting the previous and current results obtained for the representatives of coxibs, one can observe a higher negative divergence from the Couchman–Karasz prediction in the case of the ETB–acMAL binary system, as depicted in Figure 4c. Shamblin et al. [21] widely discussed a weakness in the dependence of *T_g_* on adding an excipient to a drug in comparison with that implied by the Couchman–Karasz equation. They suggested that it could happen if the total energy of the interactions between the molecules of different species was lower than that between molecules of the same species. This hypothesis has been confirmed via DFT calculations in our previous investigations of amorphous binary mixtures containing CEL or IBU with the addition of acMAL [16,22].

It is worth noting that our isothermal refractometry measurements showed that acMAL also played the role of an effective crystallization inhibitor in the case of amorphous ETB. We observed that this excipient significantly improved the physical stability of amorphous ETB in the binary mixture with a 50 wt% acMAL content. This correlates well with the earlier-suggested molecular mechanisms of the physical stabilization of amorphous CEL in mixtures with acMAL [16], as well as the physical stabilization of amorphous pure ETB by forming H-bonded heterodimers with ETB tautomers, which hindered recrystallization [10]. The molecular mechanism responsible for preventing the recrystallization of the amorphous ETB–acMAL compositions most likely also involved forming hydrogen bonds between acMAL and ETB molecules. As a consequence, a crystalline network based on ETB molecules could not be developed, because the population of H-bonded homodimers (ETB–ETB) would be very small in an amorphous binary mixture with a 50 wt% acMAL content, in which the population of H-bonded heterodimers (ETB–acMAL) dominated. 

**Figure 5:**
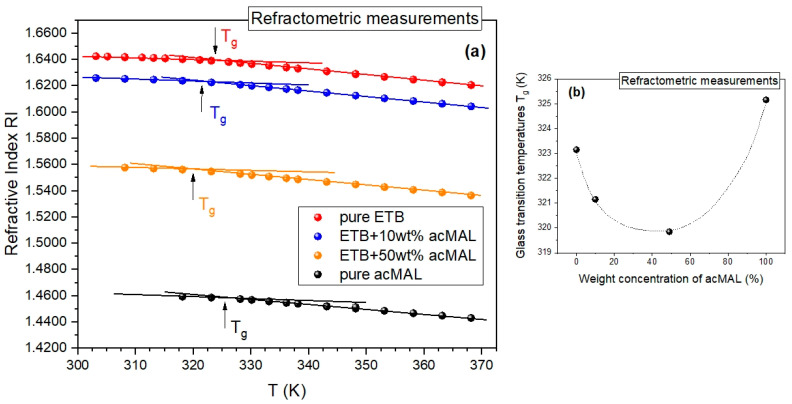
Refractometric investigations of the glass transition. (**a**) Plots of the refractive index vs. temperature for the amorphous mixtures of ETB with 10 and 50 wt% acMAL, as well as the amorphous pure components. The solid lines illustrate the method for determining the glass transition temperature at their intersection for a given material. (**b**) Plot of the refractometric glass transition temperatures vs. the percentage weight concentration of acMAL in the mixture. The dotted curve is a guide for the eyes.

The promising results of the isothermal refractometry experiments and the standard non-isothermal DSC measurements were additionally validated by analogous non-isothermal refractometry measurements carried out by cooling the binary mixtures of ETB and acMAL from a supercooled liquid to a glassy state. We were able to detect the glass transition as a change in the slope of the dependence of the refractive index on temperature, which can be compared to the symptoms of the glass transition observed in the temperature dependences of density or enthalpy. As shown in Figure 5a, it was possible to determine the glass transition temperatures from the obtained refractograms, in a way analogous to that commonly employed in such an analysis of PVT data. In Figure 5b, we present a plot of the values of *T_g_* vs. the weight fraction of acMAL, established from the refractometry experiments, which behaved non-monotonically in an analogous way to those obtained from the DSC measurements (Figure 4a). In addition, there was also no sign of recrystallization in the refractograms collected in Figure 5a, similarly to the results of the non-isothermal DSC measurements depicted in Figure 4a. This strengthened the premise about the suggested potential of the examined amorphous ETB–acMAL mixtures for pharmaceutical applications and demonstrated the usefulness of the refractometry technique in the study of the glass transition and the recrystallization process if the refractometric equipment is able to achieve a temperature range close to the glass transition of the examined system. 

### 2.3. Amorphous Binary ETB–acMAL Mixtures: Molecular Dynamics

In Section 2.2, we report the peculiar behavior of the glass transition temperatures for the amorphous mixtures of ETB with acMAL, which was obtained by means of both the DSC and refractometry measurements. Since broadband dielectric spectroscopy has been appreciated as a very robust technique for studying the molecular dynamics in many decades of timescale and various thermodynamic conditions, including the vicinity of the glass transition [17,23,24,25], we have performed thorough investigations of the ETB–acMAL compositions with the content of 20, 30, 40, and 50 wt% acMAL near the glass transition by using the BDS measurements. Using this method, we additionally validated a divergence of the dependence of the glass transition temperature of the binary mixtures on the content of acMAL from the linear prediction given by Equation (2). Since a previous study on pure ETB has led to the conclusion that there is no correlation between the glass mobility monitored by the BDS measurements and the physical stability of pure ETB at ambient pressure [10], we focused herein on the results obtained by means of the BDS technique for its binary mixtures with acMAL in a supercooled liquid state.

**Figure 6:**
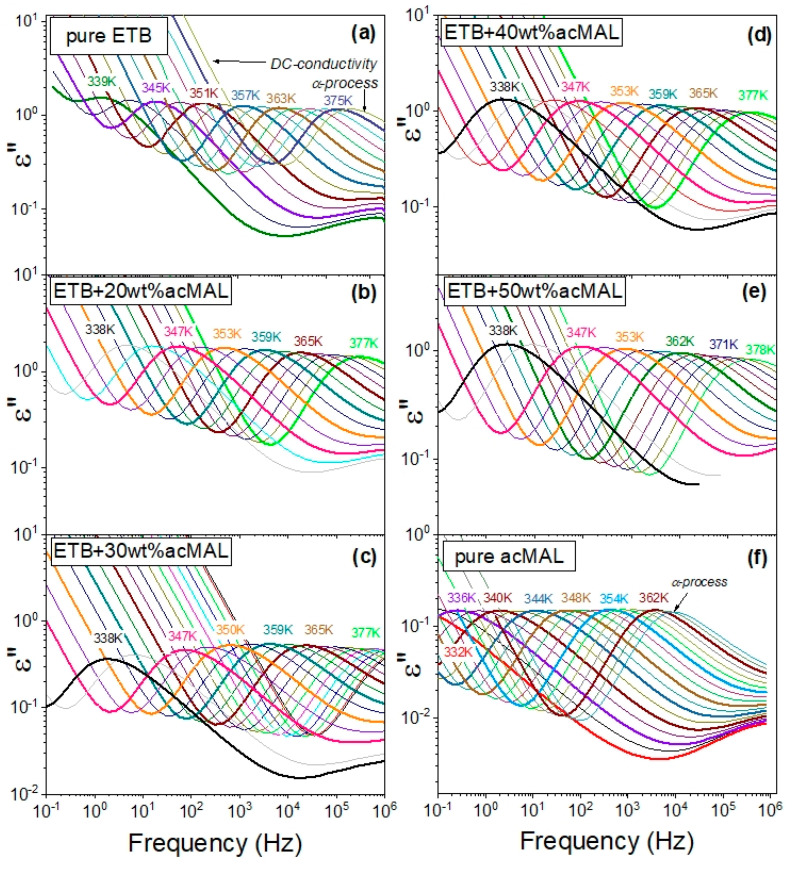
Results of the BDS measurements for the ETB–acMAL mixtures in the supercooled liquid state compared to those reported earlier for pure components: (**a**) dielectric spectra for pure ETB earlier reported [10,15] but not shown in this way, (**b**) dielectric spectra for the ETB + 20 wt% acMAL mixture, (**c**) dielectric spectra for the ETB + 30 wt% acMAL mixture, (**d**) dielectric spectra for the ETB + 40 wt% acMAL mixture, (**e**) dielectric spectra for the ETB + 50 wt% acMAL mixture, and (**f**) dielectric spectra for pure acMAL earlier reported in [16] but not shown in this way.

Figure 6 shows dielectric loss spectra measured in a wide temperature range at ambient pressure during our current research on the binary mixtures of ETB and acMAL, which were compared with the dielectric loss spectra reported earlier for the pure components ETB [10] and acMAL [16]. The first important observation made from the inspection of the dielectric loss spectra was a lack of signs of recrystallization, because there was no sharp drop in the amplitudes of the main α-relaxation process. The next behavior easily noticed in these spectra was their strong dependence on changes in temperature, as they shifted to lower frequencies with decreasing temperature, revealing a characteristic rapid slowdown in the molecular dynamics of the systems upon approaching the glass transition. In the dielectric spectra collected for the binary ETB–acMAL systems near the glass transition at temperatures above *T_g_*, one can distinguish the dc-conductivity and the structural α-relaxation process that reflects the global molecular dynamics of the systems, which is commonly considered to be relevant to the glass-transition phenomenon. For this reason, in order to determine the timescale of the global molecular dynamics of the binary systems, which is well-defined by the structural α-relaxation time *τ_α_*, we can employ the following frequency-dependent equation based on the phenomenological Havriliak–Negami (HN) term [26,27] for the complex permittivity in fitting the entire dielectric spectra:

(3) $$\varepsilon *(f)=\varepsilon^{\prime }(f)-i\varepsilon^{\prime \prime }(f)=-i\frac{\sigma_{0}}{2\pi f\varepsilon_{0}}+\varepsilon_{\infty }+\frac{\Delta \varepsilon_{\alpha }}{{\left[1+{\left(i2\pi f\tau_{\alpha }\right)}^{\xi }\right]}^{\delta }}$$

In Equation (3), the former term describes the dc-conductivity contribution, involving the dc electrical conductivity *σ*_0_ and the dielectric permittivity *ε*_0_ of the vacuum; *ε*_∞_ is the high frequency limit permittivity; and the latter term is the HN expression with the fitting parameters ∆*ε_α_*, *τ_α_*, *ξ*, and *δ*, which are widely used except for the case of the parameter values *ξ* = 1 and *δ* = 1, in order to parametrize the asymmetrically broadened dielectric loss peaks that are typical for the structural relaxation process. Such a fitting procedure followed in the domain of frequency *f* enabled us to reliably establish the structural relaxation time as the value of the fitting HN parameter *τ_α_* in the case of the examined dielectric spectra of the binary ETB–acMAL mixtures.

**Figure 7:**
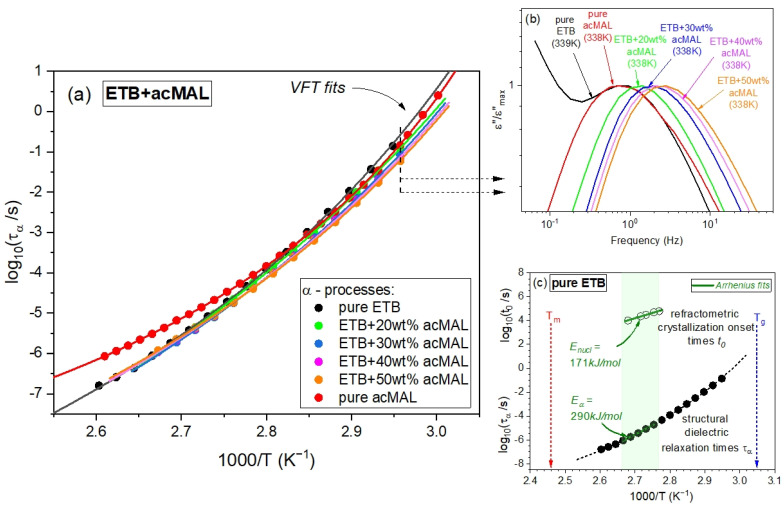
(**a**) Plot of the inverse temperature dependences of the structural dielectric relaxation times for the binary mixtures of ETB with different contents of acMAL (20, 30, 40, and 50 wt%) compared with those earlier reported [10,16] for pure components. The solid curves were established by fitting the experimental data to Equation (4) with the values of its fitting parameters collected in Table 2. The dashed and arrowed lines indicate the inverse temperature at which the spectra collected in panel (**b**) were measured. (**b**) Plot of dielectric spectra with normalized amplitudes for the examined ETB + acMAL compositions measured at T = 338 K, compared with those measured for pure acMAL at T = 338 K and pure ETB at T = 339 K. (**c**) A comparison of the recrystallization and structural relaxation timescales (open and solid points, respectively) as well as the activation energies for the nucleation and structural relaxation processes (*E_nucl_* and *E_α_*, respectively) in the temperature range available for the refractometric isothermal recrystallization experiments, at about the middle between the melting and glass transition temperatures (*T_m_* and *T_g_*, respectively).

The temperature effect on the timescale of the global molecular dynamics near the glass transition and the related glass transition temperatures determined from the BDS measurements can be conveniently analyzed by using the inverse temperature dependence of the structural relaxation times, *τ_α_*, found by fitting the dielectric spectra of the binary ETB–acMAL mixtures to Equation (3). In Figure 7a, we present the dependences *τ_α_*(1/*T*) obtained herein for the ETB–acMAL compositions, compared with those reported earlier for the pure components ETB [10] and acMAL [16]. To parametrize the inverse temperature dependences of *τ_α_*, we applied the Vogel–Fulcher–Tammann (VFT) equation [28,29,30], containing only three fitting parameters, *τ*_0_, *A*, and *T_VFT_*:

(4) $$\tau_{\alpha }=\tau_{0}\exp\left(\frac{A}{T-T_{VFT}}\right)$$

which is commonly used in such analyses, including the phenomenological description of the experimental study of molecular dynamics in pharmaceutical materials. The values of the VFT parameters obtained from fitting the inverse temperature dependences of the structural dielectric relaxation times to Equation (4) are collected in Table 2 for the binary ETB–acMAL mixtures as well as the pure components. 

The high quality of the VFT fits, represented (among other things) by small uncertainties in the determination of the VFT parameter values and the fitted curves that well-matched the experimental data in Figure 7a, enabled us to employ Equation (4) to find the values of *T_g_* from the BDS measurements of the ETB–acMAL compositions and explore the sensitivity of their molecular dynamics to changes in temperature near the glass transition. A fundamental measure of the latter is the isobaric fragility parameter [31]:

(5) $$m_{p}={\left(\frac{\partial \log_{10}\tau_{\alpha }}{\partial T^{-1}}\right)}_{T=T_{g}}$$

which has been widely promoted by Angell and many other authors [24,32,33,34,35] as a key parameter that can be used to classify glass formers according to the value of *m_p_* into strong ($m_{p}\leq 30$), moderately fragile ($30<m_{p}<100$), and fragile ($m_{p}\geq 100$) materials. A consequence of the definition given by Equation (5) and the specificity of the BDS experiments is the ability to compare the fragility values obtained for different systems measured by means of the BDS technique, which are evaluated at the same timescale of global molecular dynamics. Such arbitrary conditions of determining *T_g_* from dielectric data are related to the considerable slowdown in the structural relaxation when approaching the glass transition, which becomes too slow to be monitored by the BDS measurements. For the same reason, the structural dielectric relaxation times at temperatures below *T_g_* are usually only predicted, and the glass transition temperature is arbitrarily established at an assumed timescale of the primary dielectric relaxation process. 

**Figure 8:**
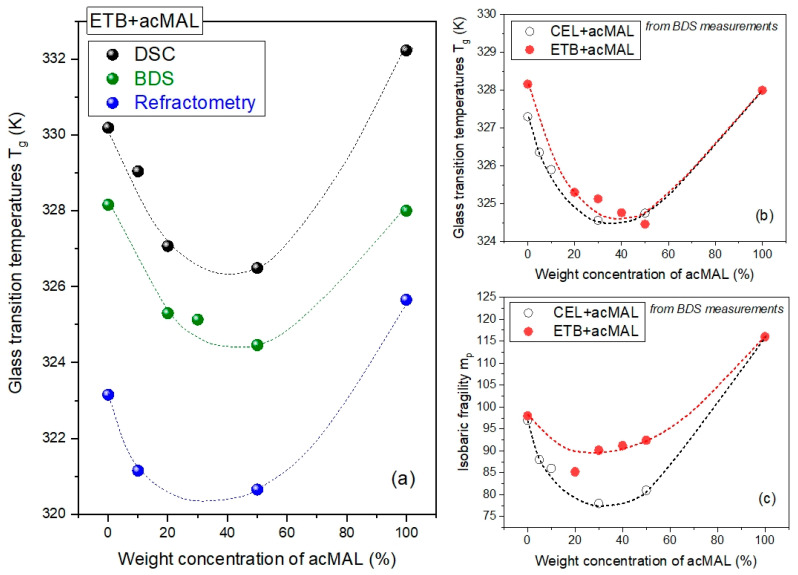
(**a**) Comparison of the temperature dependences of the glass transition temperature on the percentage weight concentration of acMAL in the binary ETB–acMAL mixtures obtained from DSC (black points), BDS (green points), and refractometric (blue points) measurements. The dotted curves are guides for the eyes. (**b**) Comparison of the peculiar behaviors of the dependences of *T_g_* on the content of wt% acMAL in the binary mixtures established herein from BDS measurements for the amorphous ETB–acMAL compositions and previously reported [16] for the CEL–acMAL mixtures. The dotted curves are guides for the eyes. (**c**) Comparison of the dependences of the isobaric fragility parameter *m_p_* on the content of wt% acMAL in the binary mixture, evaluated herein from Equation (5) based on the BDS measurements for the amorphous ETB–acMAL compositions and previously reported [16] for the CEL–acMAL mixtures.

Herein, we determined the values of *T_g_* at *τ_α_* = 100 s, which was in accordance with an assumption made earlier [16] for the amorphous CEL–acMAL compositions. By extrapolating the VFT fits to lower temperatures, at which the timescale *τ_α_* = 100 s, we found the values of *T_g_* for the examined systems based on the BDS measurements. A comparative illustration of the values of *T_g_* obtained using DSC, BDS, and the refractometry techniques is depicted in Figure 8a. The values of *T_g_* slightly differed depending on the experimental method. This is reasonable, because the measurement rates are different for each of these techniques. Additionally, during the BDS and refractometry experiments, the equilibration of the sample to a fixed temperature and the measurement at the fixed temperature take a considerably longer time than for the DSC measurements. However, it turned out that the peculiar non-monotonic dependence of *T_g_* on the content of acMAL in the amorphous mixtures with ETB was found to be independent of the applied experimental methods. This finding validates the negative divergence from the Couchman–Karasz prediction for the binary mixtures; as already mentioned in Section 2.2, this could be reasonably related to the hypothesis of the molecular mechanisms of the physical stabilization of the amorphous ETB–acMAL compositions, most likely caused by forming H-bonded heterodimers between the ETB and acMAL molecules, thus preventing the formation of the crystallization nuclei built from ETB molecules. 

Similar to the values of *T_g_* obtained from the DSC measurements (see Figure 4c), the results obtained from the BDS experiments for the amorphous mixtures of ETB with acMAL and CEL with acMAL revealed a minimum for the dependence of *T_g_* (wt% acMAL), as depicted in Figure 8b. Thus, the next reinforced conclusion is that there was no anti-plasticization effect of acMAL on either ETB or CEL. However, this was accompanied by the physical stabilization of both kinds of amorphous compositions. By invoking earlier reports on the plausible molecular mechanisms underlying the enhanced resistance of the binary CEL–acMAL systems to recrystallization [16], it should be noted that the formation of heterodimers by the CEL and acMAL molecules consisted of hydrogen bonding between the donors from the sulfonamide group of the phenyl-SO_2_NH_2_ part belonging to the CEL molecule and the acceptor sites of the acMAL molecule, which is devoid of donors. However, the ETB molecule had no sulfonamide group and the part that most likely provided donors was the methylpyridine-based ring in the tautomer, denoted as E2 in [10] and ETB 2 herein in Table 3 of Section 3.1. It was characterized by the proton transfer from the methyl group to the nitrogen atom in this ring. 

By comparing the inverse temperature dependences of *τ_α_* determined earlier for the pure components, ETB and acMAL, in the supercooled liquid state with those obtained herein for the binary ETB–acMAL mixtures, we observed some decreases in the steepness of the dependences *τ_α_*(1/*T*) in the vicinity of *T_g_* for the compositions, as depicted in Figure 7a. As already mentioned, a proper measure of the steepness is the isobaric fragility defined by Equation (5). The factor *m_p_* has been widely used to predict the physical stability of amorphous pharmaceuticals and other glass-formers. For many decades, it has been believed that the physical stability of drugs in both the supercooled and glass states is better if they are stronger materials, i.e., characterized by smaller values of the fragility parameter [13,36,37,38,39,40]. In the literature, there are known exceptions in the suggested correlation for both pure drugs and pharmaceutical compositions [2,5,10,22,41,42,43]. To verify the hypothesis in the case of the binary mixtures of ETB with acMAL, we calculated the values of *m_p_* by using Equations (4) and (5) and plotted the obtained results in Figure 8c compared to those reported earlier for the CEL–acMAL mixtures. For both kinds of binary mixtures, the dependences of the isobaric fragility on the content of acMAL behaved similarly. The values of *m_p_* initially decreased, but they began to increase starting at about 30 wt% acMAL content in the mixtures. However, the pure components were characterized by higher values of isobaric fragility. The latter indicated that the pure components had a higher global molecular mobility near *T_g_*, and it may suggest that the pure components have a stronger tendency to recrystallize than their mixtures with acMAL. Nevertheless, the increase in the values of *m_p_* observed after reaching a minimum of about 30 wt% acMAL in the binary compositions for both kinds cannot be correlated with the better physical stability of amorphous mixtures with a higher content of acMAL. The non-monotonic behavior of the fragility parameter dependence on the weight concentration of acMAL in the mixture with ETB constitutes an additional rationale [16,22,41,43,44] for the important role of specific interactions, as discussed herein hydrogen bonding, in the improvement of the physical stability of the ETB–acMAL compositions. 

By comparing the map of structural relaxation times, shown in Figure 7a, and the dielectric spectra selected at a fixed temperature near *T_g_*, presented in Figure 7b for pure acMAL and all tested binary mixtures at T = 338 K, and only for pure ETB at the nearby temperature T = 339 K, one can also observe a non-monotonic variation in the frequencies at which the dielectric loss spectra revealed their maxima with the content of acMAL in the examined systems under isothermal conditions near the glass transition. Such a non-monotonic behavior is mapped to the structural relaxation times *τ_α_* as a function of the mixture composition at a constant temperature close to *T_g_*, which reflects a non-monotonic temperature sensitivity in the global molecular mobility of the examined systems near the glass transition.

Finally, we seized the opportunity to test another suggested correlation widely discussed in the study of supercooled liquid recrystallization. This correlation relies on the classical nucleation theory, which considers thermodynamic and kinetic contributions to the crystallization process, where the latter is expected to be dominant near the glass transition if the crystallization process is governed by the diffusion [38,45]. In such a case, the characteristic timescale of crystallization should be energetically coupled with the timescale of the global molecular mobility. Since we determined the crystallization onset times *t_0_* in Section 2.1 and the structural dielectric relaxation times *τ_α_*, we were able to compare the activation energy for nucleation *E_nucl_* and the activation energy for structural dielectric relaxation *E_α_* in the temperature range, within which the isothermal crystallization refractometry experiments were carried out. This was important due to a long history of such investigations in the case of amorphous pharmaceuticals, reported, for example, in [2], which have invoked both the crystallization onset time *t_0_* and the overall crystallization characteristic timescale, where the latter was used to evaluate the activation energy for the overall crystallization process. The rationale for this study originates from classical nucleation theory, which assumes both thermodynamic and kinetic contributions to the overall crystallization process as well as to their components, including nucleation and crystal growth.

As can be seen in Figure 7c, in the considered temperature range, the inverse temperature dependence of the structural relaxation time can be also well-fitted to the Arrhenius law, which yields a linear representation of the dependence *τ_α_*(1/*T*) with $E_{\alpha }=\left(290\pm 4\right)\mathrm{kJ}/\mathrm{mol}$. Taking into account the value $E_{nucl}=\left(171\pm 7\right)\mathrm{kJ}/\mathrm{mol}$, as estimated in Section 2.1, the coupling parameter *S* between the recrystallization process and the structural dielectric relaxation can be found from the ratio $S=E_{nucl}/E_{\alpha }$, which yields a value of about 0.6 for ETB in the temperature range of 361.15–373.15 K, within which the isothermal recrystallization measurements of pure ETB have been performed herein by means of the refractometry technique. In a similar temperature range, 363.15–378.15 K, Dantuluri et al. [12] carried out isothermal recrystallization experiments for CEL by using the BDS technique, thus finding a coupling parameter value of 0.7 for the overall isothermal recrystallization. Such values for the coupling parameter indicate that the nucleation component of the examined crystallization of ETB and the overall crystallization of CEL are not solely controlled by diffusion. Nevertheless, this simple conclusion requires further considerations. It should be noted that dielectric spectroscopy, which uses probing reorientations of the permanent dipoles, cannot directly investigate the translational diffusion, and an additional problem is a decoupling that may occur between the rotational diffusion and the structural dielectric relaxation time, as described by the fractional Debye–Stokes–Einstein equation [46,47,48,49]. Thus, the decoupling phenomena observed for the mentioned rotational quantities, and also the translational diffusion and viscosity, may make the coupling parameter values difficult to satisfactorily interpret [2,50]. Moreover, it is worth mentioning that the obtained values of the coupling parameter *S* for ETB herein and CEL in [12] could have simply resulted from the temperature range available for the isothermal crystallization experiments, which usually become impossible or extremely time-consuming when approaching the glass transition. Based on the DSC measurements, ETB and CEL are characterized by the same glass transition temperature *T_g_* = 330 K, while their melting temperatures are *T_m_* = 407 K and *T_m_* = 434 K, respectively [10]. Thus, the temperature ranges within which the recrystallization experiments were performed for both drugs were located relatively far from the glass transition temperature, and they were instead in the middle of the temperature interval between the melting and glass transition points for these materials. As argued by Ediger et al. [39], the domination of the kinetic contribution to the crystallization process is actually expected closer to the glass transition, while the thermodynamic contributions to crystallization may predominate over the kinetic ones in the vicinity of the melting point. This could be the main reason for the values of the coupling parameter *S* as estimated by exploring the isothermal crystallization and the structural relaxation in the middle of the temperature range between *T_m_* and *T_g_*. 

## 3. Materials and Methods

### 3.1. Materials

Etoricoxib (ETB), belonging to a group of selective COX-2 nonsteroidal anti-inflammatory drugs (NSAIDs), is the active pharmaceutical ingredient (API) examined herein. The crystalline form of ETB, with a molecular mass of 358.8 g/mol and 98% purity, was supplied from Sigma-Aldrich (St. Louis, MI, USA). Beta-D-maltose octaacetate (acMAL), with a molecular mass of 678.59 g/mol, was used in the presented studies as an inhibitor of the amorphous drug recrystallization and was purchased from Iris Biotech GMBH (Marktredwitz, Germany) in the crystalline form. The chemical structures of the examined materials have been presented in Table 3.

The amorphous binary systems ETB + acMAL with different amounts of excipient (acMAL) were prepared by quench-cooling the supercooled liquid phase of the binary mixtures. First, the crystalline forms of ETB and acMAL were separately heated above their melting points, and then vitrified by the fast transfer of the melted samples from a hot plate (CAT M 17.5) onto a cooled copper plate. Then, the API in a glassy state was added to glassy acMAL in an amount needed to obtain the desired weight percentage concentration of the excipient in the binary mixture. In the next step, the sample containing the unmixed amorphous compounds was heated to a temperature of 353 K, at which both solid substances changed into the liquid phase. In the liquid state, both substances (ETB and acMAL) were thoroughly mixed for a sufficiently long time to obtain a homogeneous mixture. It should be emphasized that the temperature (T ≈ 353 K) at which the mixtures were prepared was significantly lower than the melting points *T_m_* of both substances, which ensured that they did not undergo any thermal degradation. Next, a homogeneous ETB–acMAL binary solution was vitrified by a quick transfer from a hot plate onto a cooled metal plate. Calorimetric measurements confirmed that all the prepared solutions (ETB + acMAL) with different acMAL contents were completely amorphous.

### 3.2. Refractometry

The refractive index (RI) measurements of the examined samples were carried out using a Mettler Toledo refractometer RM40 in the temperature range from 368 K to 303 K. The temperature stability, controlled with the aid of a built-in Peltier thermostat, was better than 0.1 K. The light source was a light-emitting diode (LED), the beam of which passed through a polarization filter, an interference filter (589.3 nm), and various lenses before it reached the sample via a sapphire prism, characterized by a high thermal conductivity. The measurements of RI were performed with a resolution of 0.0001. The sample, equal to about 500 μL, was put on the prism located in the center of the bottom of the covered measurement cell, the inner surface of which was the lateral surface of a cone truncated at its bottom. The inner bottom and top diameters of the measurement cell equaled about 1 cm and 10 cm, respectively. The sample covered the top of the prism and the sample thickness was usually about 3 mm. The refractometric measurements of the glass transition were carried out by registering the value of the refractive index during the cooling of the investigated supercooled liquids. Moreover, several isothermal refractometric measurements were performed on the crystallization from the supercooled liquid state as a function of time.

### 3.3. Differential Scanning Calorimetry

Calorimetric measurements of the investigated binary mixtures (ETB + acMAL) were carried out using a Mettler Toledo DSC apparatus equipped with a liquid nitrogen cooling accessory and an HSS8 ceramic sensor (heat flux sensor with 120 thermocouples). Temperature and enthalpy calibrations were performed by using indium and zinc standards. Each measurement at a given heating rate was repeated 3 times to confirm its repeatability. For each experiment, a new amorphous sample was prepared and placed in a measuring aluminum crucible, the volume of which equaled 40 μL, but it should be noted that the sample thinly covered only the bottom surface of the crucible. Thus, the sample volume was much less than 40 μL. The inner shape of the crucible was close to cylindrical (the cylinder’s base had a diameter of about 5 mm). The crucibles with samples were sealed at the top with one puncture. The thermal properties of several mixtures of ETB with different concentrations of acMAL were investigated.

### 3.4. Broadband Dielectric Spectroscopy

Isobaric measurements of the dielectric permittivity ε*(*f*) = ε′(*f*) − *i*ε″(*f*) were performed using the Novo-Control Alpha dielectric spectrometer over the frequency range of 10^−2^–10^6^ Hz and in a wide temperature range at ambient pressure. The technical details and principles of the BDS measurements by using this equipment have been exhaustively reported by Schaumburg [51]. Non-isothermal dielectric measurements of the ETB–acMAL mixtures were performed in a parallel-plate cell immediately after preparing the amorphous sample. The sample temperatures were controlled by a Quatro System using a nitrogen gas cryostat. The temperature stability was better than 0.1 K. The examined samples were placed between the steel electrodes of a 15 mm diameter capacitor with a fixed distance between electrodes (0.1 mm), provided by fused silica spacer fibers supplied by Novo-Control, where the sample volume was slightly less than 18 μL.

## 4. Summary and Conclusions

We thoroughly examined the recrystallization tendency of the amorphous anti-inflammatory drug etoricoxib, which has been earlier considered to not undergo recrystallization at ambient pressure. For the first time, we have shown that ETB recrystallizes in a supercooled liquid state during isothermal recrystallization experiments performed by means of the standard refractometry technique. To the best of our knowledge, this is the first successful application of standard refractometry for the systemic examination of physical stability and the glass transition phenomenon. Nevertheless, this experimental achievement revealed an application warning for the manufacturing of amorphous ETB, which can recrystallize not only at elevated pressure [10,15], but also at normal pressure, and this consequently requires finding a method for the physical stabilization of its amorphous form. 

The previous successful enhancement of the physical stability of amorphous CEL by mixing it with acMAL [16] has encouraged us to make an attempt at applying the same approach to improving the physical stability of amorphous ETB. The refractometric measurements of the binary mixture of ETB with the content of 50 wt% acMAL under isothermal conditions confirmed an increase in the physical stability of supercooled ETB. Thermal, refractometric, and molecular dynamic investigations of the binary compositions of ETB with different contents of acMAL have enabled us to formulate a hypothesis about the molecular mechanisms that may govern the physical stability of amorphous ETB–acMAL mixtures. All experimental methods used in this work (BDS, DSC, and refractometry) have provided evidence for the non-monotonic behavior of the dependence of the glass transition temperature *T_g_* on the content of acMAL in the binary ETB–acMAL mixtures, manifested by the minimum of this dependence, which significantly differs from the prediction expected in the case of a dominant anti-plasticization role in the physical stabilization accompanied with no specific interactions. Taking into account the experimental results and considering the previously discussed molecular mechanisms of the physical stabilization of amorphous pure ETB as well as amorphous binary CEL–acMAL mixtures, we may postulate that the formation of hydrogen-bonded heterodimers between ETB and acMAL molecules underlies the improvement of the recrystallization prevention in the amorphous ETB–acMAL compositions. Our hypothesis is that the plausible formation of hydrogen bonds occurs between acMAL molecules with one of the ETB tautomers containing a methylpyridine-based ring modified by a proton transfer from the methyl group to the nitrogen atom, which favors the formation of the H-bonded heterodimers between ETB and acMAL molecules, dominating over the homodimers of ETB molecules that would have facilitated the crystal nuclei aggregation. In the future, this molecular picture requires verification by performing, for instance, combined DFT and molecular dynamics simulations. 

Finally, we have verified the widely discussed correlations between isobaric fragility and the tendency to recrystallize, as well as the crystallization onset times and the structural dielectric relaxation time in the case of the amorphous ETB–acMAL compositions. Consequently, we have obtained the next examples of invalid predictions of physical stability based on the fragility parameter value. However, we have found a reliable coupling degree for the crystallization process and structural dielectric relaxation, suggesting that the nucleation component of the crystallization process is only partially governed by kinetic factors in the temperature range of the isothermal recrystallization experiments, which is plausible for ETB in the middle temperatures between the glass transition and melting points. 

## Figures and Tables

**Table 1:** Calorimetric glass transition temperatures, *T_g_*, obtained at a heating rate of 10 K/min for the amorphous ETB–acMAL mixtures compared to those earlier reported for the pure components.

Material	*T_g_*/K
pure ETB	330 ^(a)^
ETB + 10 wt% acMAL	329
ETB + 20 wt% acMAL	327
ETB + 50 wt% acMAL	326
pure acMAL	332 ^(b)^

^(a)^ Data taken from [10]; ^(b)^ data taken from [16].

**Table 2:** Values of the VFT parameters, found by fitting the experimental dependences presented in Figure 7a to Equation (4).

Material	VFT Parameters		
	log_10_(*τ_∞_*/s)	*A*/K	*T_VFT_*/K
pure ETB	−16.8 ± 0.5	2730 ± 190	265 ± 4
ETB + 20 wt% acMAL	−19.3 ± 0.7	4000 ± 360	244 ± 5
ETB + 30 wt% acMAL	−17.6 ± 0.5	3200 ± 220	254 ± 4
ETB + 40 wt% acMAL	−16.7 ± 0.4	2880 ± 150	258 ± 3
ETB + 50 wt% acMAL	−16.0 ± 0.3	2635 ± 95	261 ± 2
pure acMAL	−12.3 ± 0.1	1400 ± 20	285 ± 1

**Table 3:** Chemical structures of the investigated compounds.

API	Crystallization Inhibitor
**Etoricoxib (ETB)** **—two tautomers (ETB 1 and ETB 2)** (M_w_ETB_ = 358.8 g/mol)	**Octaacetylmaltose (acMAL)** M_w_acMAL_ = 678.59 g/mol
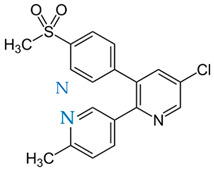 **ETB 1** 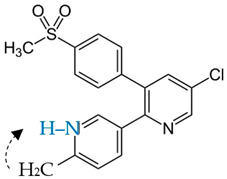 **ETB 2**	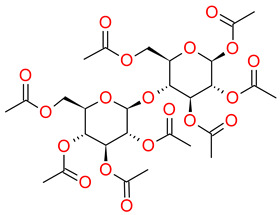

## Data Availability

The data presented in this study are available from the corresponding author upon request.
